# The Superiority of High-Fusing Porcelain for Dental Purposes

**Published:** 1904-06

**Authors:** Herbert L. Wheeler

**Affiliations:** New York


### THE SUPERIORITY OF HIGH-FUSING PORCELAIN FOR DENTAL PURPOSES.[1]

BY DR. HERBERT L. WHEELER, NEW YORK.

THE terms used in the title of this paper are somewhat arbitrary, and express the meaning which I desire to convey to you only in a general way. The manufacturers of low-fusing materials have been pleased to call their product enamels, etc., and to consider any material which baked below the melting point of gold as low-fusing; and any material with which gold could not be used as a matrix, because the heat required to bake it was greater than the fusing-point of gold, as high-fusing material. It is probably impossible to state just where the line should be drawn which separates a high- from a low-fusing porcelain, except so far as I am acquainted with the low-fusing product at present obtainable for dental purposes.

There seems to be two methods of obtaining a powder that will fuse at a temperature so far below the melting-point of gold that there is no danger in using this metal as a matrix. One of these methods seems to produce a real porcelain in that it contains ingredients which are not changed or fused in the baking process. This quality is secured by the use of artificial flux, either in the mix with the color frit, or by adding quantities of pulverized glass of a variety that contains lead oxide, to lower the degree of temperature required to cause the fusible parts of this material to melt. This

---

[1] Read before the Academy of Stomatology, Philadelphia.

particular quality increases the liability of the color to flee and the probability of gassing or bubbling. Experience has demonstrated that this substance is sooner or later effected unfavorably by the fluids of the mouth, a discoloration or a disintegration of the surface of an inlay made from this substance taking place in many instances. The other process is somewhat different and is, I think, made in the following manner: Felspar, which will fuse at a low temperature, is procured by either an admixture of different varieties or by recalcining and rebaking a felspathic rock several times; or perhaps both methods are used to secure the desired results. For it is well known that the baking of porcelain and repulverizing lowers the fusing-point without correspondingly producing loss of color in overbaking; and the porcelain enamel on the market in some of its qualities seems to indicate that this process may have been used in its manufacture. Except that the color flees so quickly after the glazing-point has been reached, I doubt if the recalcining process is used to any extent in the making of this material. While there may or may not be oxide of lead used in this latter class, I still consider it as a glass rather than a true porcelain; because, as near as I can judge by careful observation, all the component parts are fused in the baking process.

The most representative product now on the market of the first class above mentioned is the Ash & Sons low-fusing mineral bodies. The only representative of the second class, so far as I know, is the Jenkins enamel. If one is to use a low-fusing material, it seems to me the Ash & Sons product is quite superior in many respects. Its color is less apt to flee in the baking process, it can be shaped to any desired form with much greater certainty of retaining that form when baked, and the color frits are so well mixed and the substance of such a quality that it forms a much more translucent inlay than does the Jenkins enamel. On the other hand, in negative qualities the Jenkins enamel seems to be less desirable, because of the general opaque appearance, its tendency to spheroid or ball up in the baking process, its great shrinking tendency, and the temperature at which the color will flee and the number of degrees between which glazing takes place and loss of color occurs, being less than any ceramic material used for dental purposes to-day. However, my belief is, after several years observation, that no low-fusing material for porcelain inlays has ever fulfilled the hopes of those that have been induced to use it, when subjected to the test of time. The Ash & Sons material has been with us a long time; and who has not seen cases where a roughening or disintegration of the glazed surface has occurred, resulting in a changed and blackened appearance of the filling, which has entirely ruined it from an æsthetic point of view. Take also the Jenkins enamel, the formula for which has been constantly changed, if one can judge by the oft-published statements of the manufacturer who produces it. Even with this constant change, who has not seen instances of roughened surfaces, more or less disintegrated and blackened, of inlays made of this material, which is always perfect and yet is ever being perfected.

Now, I do not wish to say that low-fusing porcelain or enamels are useless. But what I do wish to impress upon you is this: that in a general way, to lower the fusing-point of an enamel means to increase the ingredients that contain alkaline salts, and that in a general way, in proportion as the alkaline parts are increased, the strength of the enamel and its ability to withstand the solvent action of the fluids of the mouth is decreased. Probably many of you know that the clay kaolin, which is one of the materials used in making porcelain, is nothing more nor less than disintegrated felspar, in which the most of the alkaline constituents, such as potassium, sodium, magnesium, etc., have been washed out by being exposed to the elements during the ravages of time. If, then, felspathic rocks are utterly destroyed or disintegrated by the action of the wind and rain, are there not great possibilities for disintegration in the fluids of the mouth, when you come to consider that in all low-fusing bodies the alkaline, or soluble, proportions are greatly increased by artificial means? And I do think that those of us, who have the best interests of our patients at heart, will be very cautious about the use of these enamels, until their permanency, under all reasonable conditions, are much better established, than they are at present.

Another very disappointing place in which I have had the misfortune to use the Jenkins enamel several times is this: it is my habit to rearrange porcelain crowns, facings, etc., by grinding in some places, adding contour in others, by means of baking on new porcelain, in order to reproduce the individual peculiarities of the teeth in different patients. Now, it has been my experience in using the Jenkins enamel for this purpose that it invariably crackled badly. The reason for this is, I think, the difference in contraction between this body and all the different bodies used in making porcelain teeth or crowns. In a good high-fusing porcelain the amount of alkaline proportions is reduced to the minimum quantity required to obtain a homogeneous mass; also at the present stage of electric ovens it is necessary to have a material that does not fuse much above 2400° F., for the reason that both the clay and the platinum will not stand a higher heat than this for any length of time without causing disaster to the oven.

There are, as you probably know, certain chemical changes that take place during the baking process of porcelain, and these processes can be varied somewhat by changing certain qualities of the ingredients without changing the proportions; that is to say, it would be quite possible to have the formula of two bodies apparently identical, but the silica in one might be much more finely powdered than in the other. The result would be that the one with the finely powdered silica would fuse at a lower temperature, the color would more readily disappear, and the strength would, I think, not be as great. On the other hand, it might be possible to leave the silica so coarse and put in such a small proportion of felspar that the glazed surface would not be perfectly smooth. It is my opinion that a material of this latter quality might have greater strength than the more homogeneous and more artistic product with the finely pulverized silicon. The silica in high-fusing bodies prevents shrinkage or retards it, and overcomes the tendency to warp or change shape noticeable in the more easily baked lower-fusing bodies. A fair amount of this ingredient gives that quality, so necessary in some cases, that admits of building out corners to a sharp edge, or of producing those concavo-convex inlays sometimes so necessary for the proper reproduction of the natural shape of a tooth at the cervical margin of the crown. This material also seems to increase the strength of porcelain. It is this ingredient that breaks up the rays of light in a porcelain body and still permits of translucency. Those low-fusing enamels which do not contain silica would be transparent like glass were it not for coloring-matter or the quality of clay used, which seems to prevent translucency to some extent and to produce a slight opacity. Then, too, the greater stability of the high-fusing material permits the work being done with much less time spent in close observation of the baking process; but the most valuable quality of a high-fusing porcelain lies in its greater resistance to chemical action by the fluids of the mouth and its less probability to disintegration and roughening of its surface.

That a translucent substance is necessary for a resemblance to the natural appearance of a tooth seems to be generally agreed upon. If it were not for this, manufacturers would doubtless be producing and selling teeth made of a material quite similar to the Jenkins enamel, especially as this low-fusing material, so far as the cost of ingredients goes, would be much less expensive than the high-fusing compounds already in use. It seems rather astonishing when one considers that inlays have been made now and then since 1857 of high-fusing material, and so far as I know there has never been a complaint of destructive action of the fluids of the mouth upon this material. On the contrary, I do not know and never have heard of a low-fusing enamel of which there were not reports from various sources of its disintegration and destruction from the action of the fluids of the mouth.

The first porcelain inlays I have seen a record of were described by Dr. A. J. Volck in the *American Journal of Dental Science* for July, 1857. These were set by packing gold around the inlay between it and the cavity margins. Down to the present time this method has remained somewhat in vogue, and I have, within two years, seen the tip of a central incisor restored by Dr. J. Morgan Howe twenty or more years ago, and the porcelain and fastenings were still apparently perfect. In spite of its great disadvantages, this method of grinding already baked porcelain to fit a cavity has some things to commend it, especially to the beginner and the man who has time. The material itself is usually of superior texture to the average inlay made in a dentist's office, because it is made of higher-fusing material and is more skilfully baked and annealed than most inlays, in consequence of having been made by the skilled hands employed by the manufacturer.

The present method of making porcelain inlays by baking in a matrix which has previously been accurately fitted to the cavity was, I think, originated by William Rollins, of Boston, and first described by him in a paper read before the Society of Oral Science, in June, 1880. Dr. Rollins also originated the gas furnace for porcelain work. His ideas seem to have been seized upon by Dr. Land and exploited as his own; he also had them patented.[1]

[1] Independent Practitioner, June, 1888.

In speaking upon the history of porcelain, in a paper read by me before The New York Institute of Stomatology, last year, and published in the May, 1903, International Dental Journal, I relied somewhat upon an article of Dr. Bruck's, which was translated by Dr. Jenkins, for my data, and I find, upon a more careful examination of the facts, that the statements of Dr. Bruck are quite inaccurate or lack any published record to support them.

About the strength of the various porcelains and enamels upon the market, the reckless assertions of the superior strength of low-fusing enamel stimulated me to make a few experiments for myself, and while I have only made a beginning, they serve to show that the low-fusing products are decidedly inferior in the matter of strength. I secured these results by baking two platinum pins in a block of porcelain, each piece being made in the same mould, and subjecting them to a tensile strain until the block of enamel broke. The power required to break them was averaged up as I repeated the experiment from four to eight times, the results in pounds being as follows: Jenkins enamel, 32½; Ash & Sons low-fusing, 30½; Ash & Sons high-fusing, 45; this latter result was so high because of one remarkable block which was mixed with water instead of the fluid furnished by Ash & Sons, which stood a strain of 60½ pounds. The consolidated inlay material which was originally made by Mr. Whiteley averaged 39; the material now made by Mr. Whiteley, 41; the Brewster enamel, 19; S. S. W. material, 33½. A block of the very high-fusing body made by Dr. Parker, of Boston, would not break, the pins breaking first, leaving the body intact.

I was so firmly impressed, by watching the fusing in these cases, that the fusing-points given by Mr. Hammond in my original paper were erroneous, that I decided to try them for myself. In order to more closely observe the fusing process in high-fusing material I am accustomed to using colored glasses to protect my eyes. I obtained the following results, though I may say the Jenkins enamel was over-baked, lost color, and was stronger as a result. The degrees are Farenheit. Jenkins enamel, 1552°; Ash & Sons low-fusing, 1580°; high-fusing, 2084°; Consolidated, 2084°; Whiteley's, 2228°; Brewster's, 2084°; S. S. W., 2228°; Parker's, 2588°. A special lot sent me by Mr. Whiteley fused at 2264° and held its color in the baking process remarkably well. This also stood a strain of forty-eight pounds in the tensile tests.

In the matter of shrinkage the Parker body showed the least and stood up well at the corners. S. S. White's and the Whiteley bodies came next in these qualities. The Consolidated, Ash & Sons high-fusing, and Brewster's were next, then Ash & Sons low-fusing, and the greatest shrinker of all was the Jenkins enamel; this also showed the least tendency to retain its form when baked.

It is to be borne in mind that in the matter of strength much depends upon a proper cooling or annealing. *You can not bake a porcelain in a Bunsen flame, leaving it unprotected from the atmosphere of the room, and get good results where any resistance or strain is to be borne.* The very ease and carelessness with which low-fusing material is baked is against it because the weaknesses of structure thus produced are not immediately visible. There is no good reason why the manufacturers should not furnish an inlay material as good and as high-fusing as their teeth, except the one previously mentioned,—viz., that most furnaces will not stand the strain, though I have reason to believe the Hammond will for a time, at least. Some few members of our calling have been securing this material by breaking up teeth, reducing them to a powder, and rebaking, and I am informed from an authoritative source that the S. S. White Company are now making teeth of their enamel and without pins to sell for this very purpose. I really think that thus far this provides the very best method I know of for securing desired color and a superior quality and strength in the inlay.

